# 

**Published:** 1891-11-07

**Authors:** 


**Notices of Births, Deaths, and Marriages,** are inserted in this column at a charge of 2s. 6d. for an announcement not exceeding four lines.

### NOTICE TO CORRESPONDENTS.

All MS., Letters, Books for Review, and other matters intended for the Editor should be addressed THE EDITOR, THE LODGE, PORCHESTER SQUARE, LONDON, W.

The Editor cannot undertake to return rejected M.S., even when accompanied by stamped directed envelope.

**Notice to Advertisers and Subscribers.**

All Advertisements, Orders for Copies of THE HOSPITAL, and Business Communications should be addressed to The Publisher, *not to the Editor*, THE HOSPITAL, 140, Strand, W.C.

The Cost of Subscription, post free, is as follows:—Per week, 1½d.; per quarter, 1s. 8d.; per half-year, 3s. 3d.; per annum, 6s. 6d. Foreign:—Per annum, 8s. 8d.; payable, in all cases, in advance.

**"Burdett's Hospital Annual," 1891—1892.**

Third year of publication. 600 pp. Boards, gilt lettering. A most complete guide to British and Colonial Hospitals, Dispensaries, Nursing and Convalescent Institutions, and Asylums. Price 3s. 6d. Published by THE HOSPITAL (Limited), 140, Strand, W.C.

**NOTICE.—The Index for Vol. X. (ending September, 1891), is on sale at the Office of this Journal, price 2½d., post free.**

### GENERAL CHARITIES.

[This Column is devoted to an account of work of charitable institutions other than Medical Charities. The Editor will be glad to receive reports or news of work at 140, Strand. *Philanthropy* should be plainly written on the envelope.]

The Secretary to the British and Foreign Sailors' Society will shortly visit St. Petersburg in order to make the necessary preparations for the establishment of a Sailors' Institute in that port, of which the Empress will be patroness.

A feature in the new "Review of the Churches" will be a series of articles by Archdeacon Farrar, entitled "The Great Philanthropics," which will deal with our chief charitable institutions. The first of the series is devoted to Dr. Barnardo's Homes.

We have on several occasions drawn attention to the good done by some of the rescue societies and orphanages towards the reduction of crime in this country. Prisons are not economical institutions, and it must be gratifying to the supporters of these various rescue societies to see the gradual and steady diminution in the prison population which has taken place in recent years, and which is no doubt due, if only indirectly in some degree, to the fact that these societies transform likely criminals into useful citizens. There is, therefore, every reason for according a generous support to such societies.

Sir Donald Currie, M.P., will preside at the 227th anniversary festival of **THE SCOTTISH CORPORATION**, to be held at the Freemasons' Tavern, on St. Andrew's Day, November 30th. The expenditure last year exceeded the total income by £420, and since then the demand on the funds of the Corporation has been great, and is steadily increasing. There are nearly 200 pensions, of which 161 are of the value of £12 a-year, the remainder varying in value from £6 to £25, distributed amongst natives of Scotland, who have resided at least 12 years in London, and who must be over 65 years of age.

At the recent annual meeting of the **ROYAL SEAMEN AND MARINES' ORPHAN SCHOOL**, Sir Frederick Fitz Wygram, M.P., expressed his regret and that of others, that the institution is more of a charitable than a self-help organization. So far as he understood it, the society placed rather a premium on improvidence than on providence and thrift. In this respect it unfortunately does not stand alone; it is the fault of most charities that they tend to sap self-reliance, and it is a question whether most, if not all such institutions would not be better dispensed if it were made a condition in ordinary cases that those who received benefit from a society should contribute to its funds.

Miss Bromby, of St. John's Vicarage, Bethnal Green, deserves every encouragement in her practical attempt to brighten the lives of her fellow citizens. The industrial clubs which she has established bid fair to be a great success, if only sufficient funds are forthcoming to enable her to carry on and extend her work during the winter months—the time when the clubs are most needed. Lads are instructed at these clubs during the evenings in wood carving, wrought ironwork, repoussé brasswork, embossed leather ornamentation, and other interesting crafts, and it is found that they much prefer to spend their evenings in this profitable manner to loafing about street corners. Over ninety pupils have already gained their instruction in the clubs, of whom many have become instructors in various centres. It is hoped that during the coming winter the industries will be spread amongst girls as well as boys, but more funds are needed, and more purchasers of the manufactured goods in order to accomplish this desirable end.

As the lease of the present house of the **ASSOCIATION FOR PROMOTING THE GENERAL WELFARE OF THE BLIND** is expiring, the Committee have secured a site in the Tottenham Court Road. Owing to the increasing trade of the Institution, a fact which must be gratifying to its supporters, the Committee are anxious to build premises and workshops on such a scale as will enable them to take an increased number of blind workers and pupils, who are constantly applying. The late foundress of the association, Miss Gilbert, daughter of Bishop Gilbert, of Chichester, who was herself blind, was, we believe, anxious to have good and adequate permanent premises, and the Committee now appeal to the public to help them to carry out her wishes. The object of the Association is to teach trades to the adult blind, give them employment in the workshops of the Institution and at their own homes, and ensure a market for the sale of their work. The trades taught are basket and brush-making, chair-caning, sash-line making, and wood chopping, and it is proposed to add mat-making and mattress-making as soon as the new premises are completed. No workers are ever discharged, except for misconduct. Upwards of £560 is annually paid in gifts and pensions to aged and infirm blind, and the amount paid in wages exceeds £2,000 a-year. Pupils admitted by the Committee are expected to guarantee a weekly payment of 6s. for basket-making, and 4s. for all other trades taught. As a pupil learns a trade, this weekly payment is reduced if the work is saleable. All the blind have to find their own board and lodging, so that the Institution is essentially one for the promotion of self-help. A list of prices of the various articles made either at the Institution or at the homes of the blind can be had on application to the Manager, Mr. A. Jennings, 28, Berners Street, W.

### SCRAPS AND GLEANINGS.

A Serious earthquake in Japan has resulted in 3,000 deaths.

Typhoid has spread from Deptford to Lewisham and Rotherhithe.

The Hospital Sunday Fund Collection at Wisbech, has produced £25.

Hospital Sunday collection at Brighton has fallen short of £100 of the amount subscribed last year.

The Deptford medical officer, Dr. Roberts, reports 149 new cases of typhoid in the district during the past fortnight.

The report for the past year of the Nottingham General Hospital shows that legacies to the amount of £1,340 have been received.

It is definitely decided to erect a Cottage Home for aged widows at Albany, Surrey, in memory of the late Duchess of Northumberland.

The Duchess of Albany opened a bazaar for the sale of work in aid of the Whitechapel Working Lads' Institute, at Prince's Hall, Piccadilly.

The Central News says that business in Melbourne is greatly interfered with on account of the number of people suffering from influenza there.

The Hospital Sunday collection at Birmingham has reached the sum of £3,324, not including a large number of donations for specified institutions.

The demonstration held in honour of the opening of the Dundee Hospital is the cause of much dispute and dissatisfaction between the Trades Societies and Parochial Board.

Dalziel's Brooklyn correspondent states that a policeman named Mullinam went insane on Monday, and struck twenty persons with his truncheon, injuring some of them seriously.

Attention is called to the fact that out-patients are in the habit of tipping the porter on visiting days at the hospital as the only way to avoid being kept waiting a very long while.

The choir of the Guild of St. Cecilia have been trying the effect of soft music as a soporific in the temperance hospitals with good effect. The patients all testified to the soothing influence.

We are glad to hear that the Worcester Infirmary has become affiliated with the Royal National Pension Fund for Nurses, and will expend not less than £30 per annum for the benefit of its nurses.

At Ashford 11 anti-vaccinators were summoned last week for refusing to have their children vaccinated. The defendants included a mechanic named Hayward, who has been proceeded against upwards of 30 times.

A serious epidemic of influenza has broken out again in the mining districts of Cornwall. Redruth is particularly affected, many prominent men having been attacked, and the doctors report large numbers of cases.

The institution in question is one of the most deserving of East-end charities. It provides home comforts and club advantages that poor working youths between 13 and 18 years of age cannot otherwise enjoy.

The Council of Charing Cross Hospital announce that Mr. Alderman Evans, the Lord Mayor-Elect, will preside at the triennial festival dinner at the Hotel Metropole on November 17. A sum of £30,000 is appealed for.

A bazaar in aid of the Southsea Crèche was held last week in that town. The creche takes in children as boarders when their mothers are incapacitated from caring for them. The usefulness of the institution is widely recognised.

A bazaar was held at Clifton on the 28th ult., in aid of the Bristol Eye Hospital. It was opened by the Duchess of Beaufort, who afterwards visited the hospital. It is expected that a sum of not less than £800 will be realised.

The work of rebuilding the Royal Edinburgh Hospital for Children has already commenced. The children have been treated lately at a large building in Morningside, as the old premises had to be abandoned owing to an outbreak of typhoid.

Typhus fever appeared in Boulogne fifteen days ago, and has made great ravages. A large number of soldiers have been attacked, and, while the hospitals are full, the barracks have been evacuated. The epidemic is attributed to the water used.

The medical authorities have lately adopted a moral standard of their own that is neither popular nor defensible. They have refused cases on more than one occasion where the police have been first on the spot, under the plea that they were in custody.

The General Hospital, Bristol, at which the Duke of Edinburgh opened a new wing last week, shows a remarkable development since its foundation in 1831. In-patients in 1833 numbered 7[illegible], and out-patients 474. In 1890 in-patients numbered 2,246, and out-patients 21,457.

The Rev. Mr. Cotton, Rector of Caogh, county Kildare, and his wife, are committed for trial on a charge of having grossly neglected children under their care in the Caogh Orphanage. The evidence, it is said, revealed a most harrowing story of neglect and starvation.

The Manchester Southern Hospital for the Diseases of Women and Children held its annual meeting last week. The hospital has done good work throughout the year, and especial efforts are to be made for the continuance of the same, for which a larger income is necessary.

Last week a deputation from the Tydesley and Leigh collieries paid a visit to the Wigan Infirmary with a view to forming an association with the institution. A separate hospital was thought of, but an arrangement with an existing establishment was thought more desirable.

The Belfast Royal Hospital was attacked by the City Coroner on the score of foul atmosphere. The year's report, by showing that the death-rate, especially in surgical cases, is one of the lowest in the United Kingdom, shows the accusation to have been exaggerated, to say the least.

The Emir of Bokhara has had several consultations of late with Russian medical men concerning the prevalence of leprosy in his dominions, especially in Bokhara, which may, indeed, be considered as the hotbed of leprosy in Central Asia. The Emir has decided upon the foundation of a special hospital.

The resources of the Leicester Infirmary are being much taxed to provide accommodation for the large number of typhoid cases seeking admittance. The infirmary heating apparatus is out of order, and alterations are required to the post mortem room and mortuary. £1,500 seems rather a large sum to devote to these purposes.

### The Student of Medicine.

[All communications for this Supplement should be addressed to The Editor of The Hospital, at 140, Strand, with the words, "Students' Column," written in the left hand top corner of the envelope.]

### SYPHILIS: ITS TRANSMISSION AND TREATMENT.

(*Concluded.*)

By C. Gordon Brodie, F.R.C.S., Assistant Surgeon to the North-west London Hospital; Senior Demonstrator of Anatomy, Middlesex Hospital.

*Abstract of a paper read before the Middlesex Hospital Medical Society.*

The treatment of syphilis may be divided into the general, hygienic, and the special medicinal. 1. General. Bearing in mind that the disease has a debilitating tendency, and the class of people in whom it runs riot, are those whose mental and physical strength is undermined by excesses; the first thing to do is to improve both, to maintain or place both bodily and mental vigour upon a higher standpoint. Firstly, the most absolute cleanliness must be enforced, especially with regard to secretions; towels, &c., must be rigidly kept apart from those who may be brought into contact with the patient. During the period of eruption a good tub daily will be of benefit, with the friction of a rough towel after to stimulate the circulation in the skin, and clear away all the débris. Add to this a Turkish or hot tub once a week, but bear in mind that many persons cannot stand the former. Look to the teeth, and cleanse away tartar, and a slightly astringent mouth wash often proves of service. Regular exercise should be enjoined, sufficient to send a good glow through the system, but not to tire the patient; during the primary sore, however, it is well to bear in mind that excessive walking has been known to cause suppuration of the inguinal glands. As a mercurial course is to be prescribed, the patient ought to guard carefully against chill, and light warm flannel clothing should be worn next the skin. The diet should be light and easily assimilated, but with regard to the beverages a patient is allowed to drink, I say at once, anything but alcohol. Of course, should the primary sore take a phagedænic turn and the patient be evidently debilitated, alcohol will be necessary for a time to help recovery, but taking this out of consideration, alcohol retards the cure, and many obstinate cases have got well when their alcohol has been broken off. I remember a case of an old lady who never "took a drop," but with a most suggestive nasal organ, who rather retrogressed than improved, until I found that the "drop" did not include her nightly "quartern" or so of gin, and when this was stopped she quickly improved. Bloxham, in a paper in the *Lancet*, says he is accustomed to tell his patients that if they wish to keep their syphilis they have only to take enough alcohol. Smoking had best be interdicted, though provided there be no sores on the mouth or tongue, an occasional pipe or cigarette will do no harm; but if sores in the mouth appear, it must be stopped at once. Let the pipe, too, be kept strictly *pro propia personis*. The ordinary daily avocation may be strictly kept to, for it will occupy the mind and take it off from a steadily progressing disease. If the patient could afford it, a long sea voyage is a grand aid in restoring the health. The special medicinal treatment which has been adopted for the abortion or cure of syphilis, the former being the one preferred by Mr. Hutchinson, may be again divided into local and medicinal. (*a*) Many suggestions have been made with regard to the treatment of the hard chancre locally. Auspite claims, that by excision he could, even when the lymphatic glands were inflamed, prevent the infection of the system; but of a list of 33 cases which he gives, however, nine had no signs, 20 were affected with secondary disease, and the rest had not been under observation long enough to report on. It seems rather absurd to suppose that erasion after the lymphatics were affected would cure the disease, and it is questionable whether the cases which had no signs have not since developed them. Against the method, we can rank the opinions of Neumann, Mariac, and also Zeissl, who definitely state that such is not the case. Jennings, in the *Lancet*, claims to have cured one case by the benzoline cautery, but this has been questioned. Until we know the exact rate of absorption of the poison, I do not think it would be fair to try these means by which experienced observers have failed. Mercury in the form of the old Lot. Nigra used to be the favourite remedy, or calomel as a powder. Now a great deal of iodoform is used both as crystals and ointment, but in private practice it is objected to by most people because of the odour. The medicinal treatment really resolves itself into the question which is the best way to give mercury—for that is the sheet anchor of our barque. It has been employed from the earliest times, but owing to the large doses which were at first employed causing speedy salivation and phagedæna of the sores, it fell into disrepute with all the leading men, who regarded it with horror, as is shown by the following story, which was told by Maggins of the case of a certain Count Mirandola, "who after suffering for nine months from the disease, during which time he had wasted to a skeleton and infected his wife and child, called on him for advice, and was told that nothing could be better than guaiacum, although it had been tried four times in vain, saying that on so exalted a personage the use of mercury was out of the question" (Berkely Hill). The terrible ravages that this disease and the misuse of mercury caused at some of the French towns in the fourteenth and fifteenth centuries were sufficiently appalling to cause the above remark. I have by me an old volume which gives a prescription "de norbo gallico," beginning with half a pound of red lead, running through some dozen ingredients, and ending with a pound of cinnabar. The action of mercury upon the system is that of an alterative and tonic, and on syphilis it has an abortive, in fact, almost a curative effect. When this drug is taken into the body of a healthy individual in minute doses, both men and animals tend to increase in weight, but in large doses it proves fatal to animals. Reyes in his "Tonic Treatment of Syphilis," states that (1) about 5,000,000 red blood corpuscles in the C.M. is a full average for a healthy adult, fair health having about 4,500,000, while anæmia rarely goes below 3,000,000; (2) mercury diminishes the number of red blood cells, especially when given in hospitals; (3) syphilis decreases the number as well; (4) mercury alone or with iodide of potassium in syphilis increases the number of red cells and maintains them at a high standard. When given by the mouth it usually stated to be combined with an albumen, while by the skin usually retaining its metallic form. It is stored chiefly in the viscera and found most abundantly in the liver and spleen, being less in quantity in the brain, lungs, and kidneys. Its elimination takes place via the kidneys and intestines, a very small proportion only being carried to the tissues. Given by the subcutaneous method the quantity eliminated is greater than by the mouth, but less is thrown out when given by inunction. Bloxam states that of all the subcutaneous injections the biniodide was the slowest secreted, not appearing in the urine until the tenth injection. The drug may be used in three different ways: 1. By inunction, from a scruple to a drachm of the ointment being rubbed well into the soft skin over the inner sides of the arms, thighs, and flanks every night, and a flannel roller applied round the part to prevent friction, a most necessary precaution being never to rub it into the same part twice running, but to use each in rotation, to avoid the possibility of raising a sore. The skin should be well washed previous to the application, and the rubbing should last about ten minutes to a quarter of an hour. A fair amount of bed-clothes should be used to favour sweating. This should last about six or seven weeks, be discontinued for another week, and then again persevered with. For infants inunction is by far the best, and may be applied as suggested by Sir B. Brodie, by taking a flannel bandage smeared with Ung. hydrargic and sewing it round the infant's abdomen. Fair and hairy people do not bear this method well. 2. By vapour. This method is less liable to salivate than any other, and is least disturbing of all to the general health of the patient. The vapour bath is a convenient form, and one of the best. It is best to begin with gr. xx. of calomel, and gradually increase up to a ʒi. 3. By means of subcutaneous injections. This has received a good deal of attention during the last few years, and the following advantages are claimed for it: 1. That a very variable time elapses between the exhibition of mercury by the mouth and its action on the disease, whereas the subcutaneous method acts directly. 2. By the mouth the action is uncertain, and apt to cause ptyalism; the subcutaneous never does. 3. When given by the mouth it is apt to produce gastric disturbances; the subcutaneous method never does, and out of 1,950 cases quoted by Bloxam one only was affected with abscess at the seat of puncture. 4. That you always know the exact amount which is absorbed. The chief injections which have been used are (*a*) calomel and glycerine 1-10, which is too bulky and causes much pain. (*b*) Sigmund advises bichloride, salt, and water, which is speedily absorbed and causes but little pain. (*c*) Bloxam, in order to combine small bulks and good results, uses bichloride, ammon. chlor.; and water; m.x. = gr. $\frac{1}{8}$. This solution keeps well, and rarely causes any unpleasant effects. There must only be one injection a week, and that must be injected deeply into the muscles of the buttocks, using each alternately. The needle of the syringe must be kept carefully cleaned and sharpened, as the solution is apt to corrode it and make it brittle. A few injections causes the primary lesion to disappear and the severe signs to be arrested, and in seven or eight weeks even the most troublesome skin affections often disappear. It is continued for at least eighteen months, the total amount which is given being about 6 grs. of the salt. 4. By the mouth. Everyone is familiar with this, and also the salts used; the ordinary haust hydrarg. perchlor., when properly handled does not often give rise to unpleasant symptoms. It really consists of the freshly formed biniodide. The salt par excellence is, however, the hyd. c. cret. given in doses of gr. i. three or more times a day, and continued over a long period of time—if the case is seen early, at least nine months. It may often be combined with the ferrum redactum, or even a grain of quinine, and if a little diarrhœa is set up a small modicum of opium will quiet down the intestinal trouble. The so-called Plummer's pill is not worth much, as it often passes out without being softened. Tittman's decoction is a favourite pre-

### THE STUDENT OF MEDICINE—(continued).

paration in Germany, and comprises sarsaparilla, aromatics, and mercury salts. It is very efficient, and is used in conjunction with a hot-air bath. For Indian work, especially where there may be malarial influence, liq. hyd. perchlor., chloride of ammonium, and chiretta bark form a good mixture. Some people hold that iodide is as good in the late stages as mercury is in the early ones; but iodine often fails to cure when used alone, while in combination with mercury it acts rapidly; alone it does not assist the healing of the initial sore, and is not so good in the early skin rashes. Possibly it acts by being a solvent on the mercury, and it is a well-known fact that salivation may occur even after the mercury has been stopped while a course of iodide is being given. A recent writer looks upon iodide as so much "white-wash," yet in spite of this it is very useful in tertiary signs, and will act like magic upon the skin and headaches, but mind, it only palliates, and requires mercury to complete the cure. In cachexic people it is extremely serviceable. Many other drugs have been lauded for the cure of syphilis, but have fallen into disuse. Of these sarsaparilla is a useful adjunct to the iodide, and helps large doses to be borne with great ease. Mr. Nunn has, I believe, had good results with the terchloride of gold, but Sigmund and others ignore its power.

### APPOINTMENTS.

At King's College Hospital, E. Distin, M.R.C.S., L.R.C.P., has been appointed house surgeon. At Noble's General Hospital, Douglas, Isle of Man, Henry Buxton, L.R.C.P. Edin., L.R.C.S., Edin., L.F.P.S. Glasgow, has been appointed house surgeon. At the Sourhall Fever Hospital, John Brown, M.D., B.S., D.Sc. Vict., has been appointed physician. At the Union Infirmary, St. George's-in-the-East, London, Marcus Marwood Bowlan, M.B. Dunelm, has been appointed assistant medical officer.

### VACANCIES.

The following vacancies are announced this week, the amount of salary in each case being given after the name of the institution:

### Resident Medical Officers.

Asylum for Idiots, Earlswood, Redhill, Surrey, Assistant Medical Officer—Salary £153, &c.

Berks Asylum, Moulsford, Second Assistant Medical Officer—Salary £100, &c.

Cancer Hospital (Free), Fulham Road, S.W., House Surgeon—Salary £50, &c.

City of London Lunatic Asylum, Stone, Near Dartford, Kent, Assistant Medical Officer—Salary £120, &c.

Dudley Dispensary, Medical Officer—Salary £130, &c.

Grantham Friendly and Trade Societies Institute, Medical Officer—Salary £150, &c.

Ripon Dispensary and Cottage Hospital, House Surgeon and Dispenser—Salary £70, &c.

Sheffield Public Hospital and Dispensary, House Surgeon—Salary £100, &c., Assistant House Surgeon—Salary £65, &c., and Junior Assistant House Surgeon—Salary £50.

### Non-Resident Medical Officers.

Dental Hospital of London, Leicester Square, Dental Surgeon and Assistant Dental Surgeon.

Hospital for Sick Children, Great Ormond Street, Medical Registrar and Pathologist—Honorarium 50 guineas.

Royal Hospital for Diseases of the Chest, City Road, Physician.

### NOTES FROM THE SCHOOLS.

### Cambridge University.

The following gentlemen have been appointed examiners for medical degrees:—Physics: Professor Thomson, F.R.S., and Mr. L. R. Wilberforce. Chemistry: Professor Liveing, F.R.S., and Mr. Pattison Muir. Biology: Mr. A. Sedgwick, F.R.S., and Mr. A. E. Shipley. Pharmaceutical Chemistry: Mr. E. H. Acton, and Mr. F. H. Neville. Physiology: Dr. Shore. Human Anatomy: Professor A. Macalister, F.R.S., and Dr. A. Thomson. Assessor to the Regius Professor of Physic: Dr. D. MacAlister.

### College of Medicine, Newcastle-on-Tyne.

The annual general meeting of the Durham University Medical College Athletic Club, was held in the theatre on Oct. 19th, Mr. Page in the chair. Present: Drs. Neasham, Howden, Gray, and about 130 members. The reports of the various sections having been read and confirmed, the balance sheet was brought forward, showing a very creditable balance of £55 1s. 9½d. Mr. Page was re-elected President by acclamation, and following representatives of the various sections were chosen:—Football: Rugby—Messrs. Alexander and Bryant; Association—Messrs. Morris and Smith. Cricket: Messrs. Lloyd-Williams and Smurthwaite. Boating: Messrs. Visser and Brewis. Tennis: Messrs. Green and Jackson. Gymnasium:

### THE STUDENT OF MEDICINE—(continued).

Messrs. Clay and Whitling. Hon. Secs.: Mr. John Braithwaite, Mr. C. R. Wood.

The annual general meeting of the Musical Society was held in the theatre of the College of Medicine on Oct. 22nd. Present: Dr. McBean (in the chair), Dr. Howden, and about 60 members. A vote of thanks to the retiring President, Dr. McBean, for his past services, having been given, Dr. Howden was unanimously elected in his place. The following representatives were then elected: Second year, Mr. Wilson; third year, Mr. Visser; fourth year, Mr. Prior; beyond fourth year, Mr. Daniel. Hon. Secs. Mr. Peacock, Mr. Hodgson.

### SCHOLARSHIPS AND PRIZES.

#### St. Bartholomew's Hospital and College.

At the examination of candidates for entrance scholarships held on September 26th and following days, the following awards were made: The scholarship, value £65, in Chemistry and Physics, to Maurice Coleman and Thomas Horder—equal; scholarship, value £65, in Biology and Physiology, to Henry T. Gillett; scholarship, value £130, in Biology, Chemistry, and Physics, to Vernon H. Blackman; preliminary scientific exhibition, value £50, to James Hussey; Shuter scholarship, value £50, in Anatomy, Physiology, and Materia Medica, to J. C. Blandford, B.A.; Jeaffreson exhibition, value £20, in Classics and Mathematics, to F. H. Nimmo. Each scholarship is tenable for one year.

### FOOTBALL.

Rugby, October 17th.

Westminster Hospital v. Tooting College.—The above match took place at Tooting on Saturday, the 17th inst. The Hospital team was a fairly representative one, the College playing mostly old students. The ball was started at 3.30 sharp, and had only been a few minutes in play when the Medicos, by a grand rush, scored their first try, Witham, their captain, getting in. The kick, from a rather difficult angle, was taken by Denne, who placed a very fine goal. The leather was restarted, and now a most exciting and fast game was carried on, the College doing all in their power to retrieve their loss, the Hospital, on their part, playing a steady, but determined game. The game till just before half-time was very even. At this moment Syle-Smith dropped a very neat goal. Scores at half-time: Hospital, five points; College, four points. At the commencement of the second half, the Hospital had the wind somewhat in their favour, which no doubt compelled the College to play a more or less defensive game, the struggle being frequently in their territory, and by a very smart run Hancock (the Hospital 100 yards runner) managed to secure a second try for the Medicos. The major point was again easily registered by Denne. After this the Tooting men seemed to wake up a bit, and on one or two occasions the Hospital had to put out all its defensive powers. At this point of the game some loose play enabled Syle-Smith to rush over the Hospital line, where he fell with Nitch-Smith, one of the Hospital Half-backs. A maul was anticipated, but the referee gave the try to the College. The kick was a failure: Soon after this no side was called, and the Medicos were left victors by ten points to six.

### EVENTS FOR THE MONTH OF NOVEMBER.

#### Medical Societies.

St. Mary's Hospital Medical Society.—November 18th: Cremation, Mr. A. R. Chater; clinical cases, Mr. F. J. Stephenson.

University College Medical Society.—November 18th: Paper by Raymond Johnson, M.B., B.S., F.R.C.S., "Histological—Circulatory System;" chairman, Bilton Pollock, Esq., B.S., F.R.C.S.

Westminster Hospital Guthrie Society.—November 12th: Meeting, at eight p.m.

#### Football.

St. Mary's Hospital,—November 7th, Past v. Present, at home; 14th, v. Bedford, at Bedford; v. London Hospital (second), at home; 21st, v. Norwood, at Norwood; v. University College School, at home; 28th, v. Wasps, at Putney; v. Wasps (second), at home.

St. Thomas's Hospital.—First fifteen, November 7th, v. R.M.C., at Sandhurst; 14th, v. Kensington, at Woodlane; 21st, v. London Scottish, at Richmond; 28th, v. Rosslyn Park, at Acton. Second fifteen, November 7th, v. Guy's (second), at Lambeth; 14th, v. Civil Service, at Lambeth, 18th, v. Bartholomew's (second), at Lambeth; 21st, v. Middlesex Wanderers (second), at Richmond; 28th, v. Streatham Park, at Streatham.

University College and Hospital.—Hospital Team, November 7th, v. Ealing Club, at Ealing; 14th, v. Aldenham Grammar School, at Aldenham; 21st, v. Clapham Rovers, at Wandsworth; 28th, v. St. John's School, at Leatherhead. College Team, 14th, v. King's College Hospital, at Wormwood Scrubbs.

Yorkshire College.—November 7th, v. Leeds Good Shepherds, at home; v. Ripon Rovers, away; 14th, v. Dewsbury, St. Matthew's, at home; v. Barnsley Congregational, away; 18th, v. Bradford Grammar School, away; 21st, Leeds A., at home; v. Pateley Bridge, away; 25th, v. Ripon Grammar School, away; 28th, v. Huddersfield A., at home; v. Knaresborough, away.

